# Development of a Web-Based Supportive Care Program for Patients With Head and Neck Cancer

**DOI:** 10.3389/fonc.2020.602202

**Published:** 2020-12-15

**Authors:** Carolyn Y. Fang, Thomas J. Galloway, Brian L. Egleston, Jessica R. Bauman, Barbara Ebersole, Marcin Chwistek, Janice G. Bühler, Margaret L. Longacre, John A. Ridge, Sharon L. Manne, Cheri Manning

**Affiliations:** ^1^ Cancer Prevention & Control Program, Fox Chase Cancer Center, Philadelphia, PA, United States; ^2^ Department of Radiation Oncology, Fox Chase Cancer Center, Philadelphia, PA, United States; ^3^ Department of Biostatistics, Fox Chase Cancer Center, Philadelphia, PA, United States; ^4^ Department of Medical Oncology, Fox Chase Cancer Center, Philadelphia, PA, United States; ^5^ Department of Speech Pathology, Fox Chase Cancer Center, Philadelphia, PA, United States; ^6^ Supportive Oncology and Palliative Care Program, Fox Chase Cancer Center, Philadelphia, PA, United States; ^7^ Department of Physical Medicine and Rehabilitation, Fox Chase Cancer Center, Philadelphia, PA, United States; ^8^ Department of Public Health, College of Health Sciences, Arcadia University, Glenside, PA, United States; ^9^ Department of Surgical Oncology, Fox Chase Cancer Center, Philadelphia, PA, United States; ^10^ Department of Medicine, Robert Wood Johnson Medical School, Rutgers Cancer Institute of New Jersey, New Brunswick, NJ, United States; ^11^ Triad Interactive Inc., Washington, DC, United States

**Keywords:** head and neck cancer, survivorship, web-based program, self-efficacy, coping, symptom management

## Abstract

Patients undergoing radiation treatment for head and neck cancer experience significant side-effects that can impact a wide range of daily activities. Patients often report receiving insufficient information during and after treatment, which could impede rehabilitation efforts; they may also encounter practical and logistical barriers to receipt of supportive care. Thus, we developed a web-based program, *My Journey Ahead*, to provide information and strategies for managing symptom-focused concerns, which may be easily accessed from the patient’s home. The purpose of this study was to evaluate patient acceptability and satisfaction with the *My Journey Ahead* program. In Phase 1, five patients with head and neck squamous cell carcinoma (HNSCC) reviewed the web-based program and provided initial feedback, which informed program modifications. In Phase 2, 55 patients were recruited to evaluate the program. Patient assessments were obtained prior to and after use of the web-based program, and included measures of psychological distress, self-efficacy in coping with cancer-related issues, and satisfaction with the website. Among the 55 patients enrolled, 44 logged in and viewed the web-based program. Participants reported high levels of satisfaction with the information received, and indicated that the website was interesting and easy to use. Older age and higher levels of self-efficacy in coping were each associated with higher levels of satisfaction with the website. In summary, the web-based program was well-received by patients, the majority of whom found it to be informative and useful. An easy-to-use web-based program, particularly for older patients who may have difficulty locating reliable evidence-based information on the internet, may be helpful in addressing survivors’ needs in symptom management and coping with cancer.

**Clinical Trial Registration:**

https://clinicaltrials.gov/, NCT02442336

## Introduction

Patients undergoing treatment for head and neck squamous cell carcinoma (HNSCC) face numerous physical, psychological, interpersonal, and practical challenges as a result of their diagnosis and treatment. Despite advances in diagnosis and management, treating HNSCC often entails considerable functional impairment including not only acute, but chronic long-term changes that impact swallowing, eating, periodontal health and oral care, speech, and social functioning (1–3). As a result, routine daily activities can become more challenging due to ongoing difficulties with speech and swallowing or impaired eating ability (4–6).

HNSCC patients often report a desire for more information during and immediately after their treatment (7), and 73% of HNSCC patients have reported receiving insufficient information (7). A perceived lack of information has been associated with post-treatment anxiety and depression (8), whereas the perception of having obtained adequate information is an important predictor of positive rehabilitation outcomes in the two- to six-year post treatment period (7). Thus, providing HNSCC patients with readily accessible information about post-treatment effects and care may enhance outcomes (9).

Several interventions have targeted HNSCC patient needs (10, 11), but barriers to participation have been identified. Specifically, requiring HNSCC patients to attend in-person sessions is a major barrier to involvement, and compliance with interventions that entail repeated in-person interactions is difficult to achieve (12). Web-based interventions offer one approach for addressing practical and logistical barriers to receiving supportive services. In addition, web-based programs are responsive to patient preferences for receiving materials and interventions that can be viewed at home, and thus may cause less burden for the patient. Hence, we developed a web-based program called *My Journey Ahead* to provide information and strategies for managing symptom-focused concerns faced by HNSCC patients who were treated with radiation. In this paper, we describe patient acceptability of and satisfaction with *My Journey Ahead*.

## Methods

### Development and Website Content

Website content was informed by our prior work (13) and guided by the theoretical framework Social Cognitive Theory (SCT) (14–17), which proposes that behaviors and skills can be learned through education and modeling. For example, a SCT-based intervention demonstrated that behavioral modeling was effective in promoting physical rehabilitation and reducing limitations in the injury recovery setting (18). Thus, the website was developed to attend to the self-efficacy expectations of patients by providing various strategies and exercises to enhance coping and improve functional abilities, and through the sharing of personal experiences from other HNSCC survivors.

Program content was developed by healthcare specialists including a radiation oncologist, speech-language pathologist, palliative care and pain management physician, physical therapist, and a clinical psychologist. Website programming and creation of content (videos, graphics, interactive tools, etc.) was produced by Triad Interactive, Inc. After the initial website content was developed, five other healthcare professionals who treat this patient population reviewed the information for accuracy and readability.

#### Program Content

The program included an Introduction section to explain the use of the website, followed by four specific units:

Unit 1: What is head and neck cancer? This unit provided information about HNSCC, including informational text, brief videos of healthcare professionals describing radiation therapy, and animations to illustrate anatomy and physiology.
Unit 2: Potential changes in swallowing and oral care. This unit described potential changes in swallowing that may occur. Proper oral care and other potential side effects of radiation therapy (e.g., xerostomia, mucositis) were also discussed. Brief videos and animations presented strategies for eating and swallowing, including personal experiences described by survivors.
Unit 3: Potential changes in speech and social interactions. This unit covered possible temporary or permanent changes in speech and the role of a speech language pathologist. Physical exercises that involve mouth and tongue movement were demonstrated using brief videos. Strategies that other HNSCC survivors have used to facilitate social interactions and effective communication were presented.
Unit 4: Coping with cancer. This unit addressed psychosocial challenges and described how other HNSCC survivors have met these challenges. Behavioral strategies, social skills training, and journaling and relaxation exercises were demonstrated using worksheets, brief videos, or audio clips.

Five patients who had completed radiation therapy within the past 12 months were recruited to review website content and provide feedback on usability, using a modified version of the guidelines suggested by Usability.gov (19). Each participant viewed the program in a private area with a study coordinator who was available to provide technical assistance. After the participant finished viewing the program, the study coordinator conducted a brief interview to obtain feedback regarding ease of use; satisfaction with the information provided; and acceptability of the visual images, graphics, and videos. Feedback from participants regarding each of the four units is briefly summarized below.

**Figure 1:**
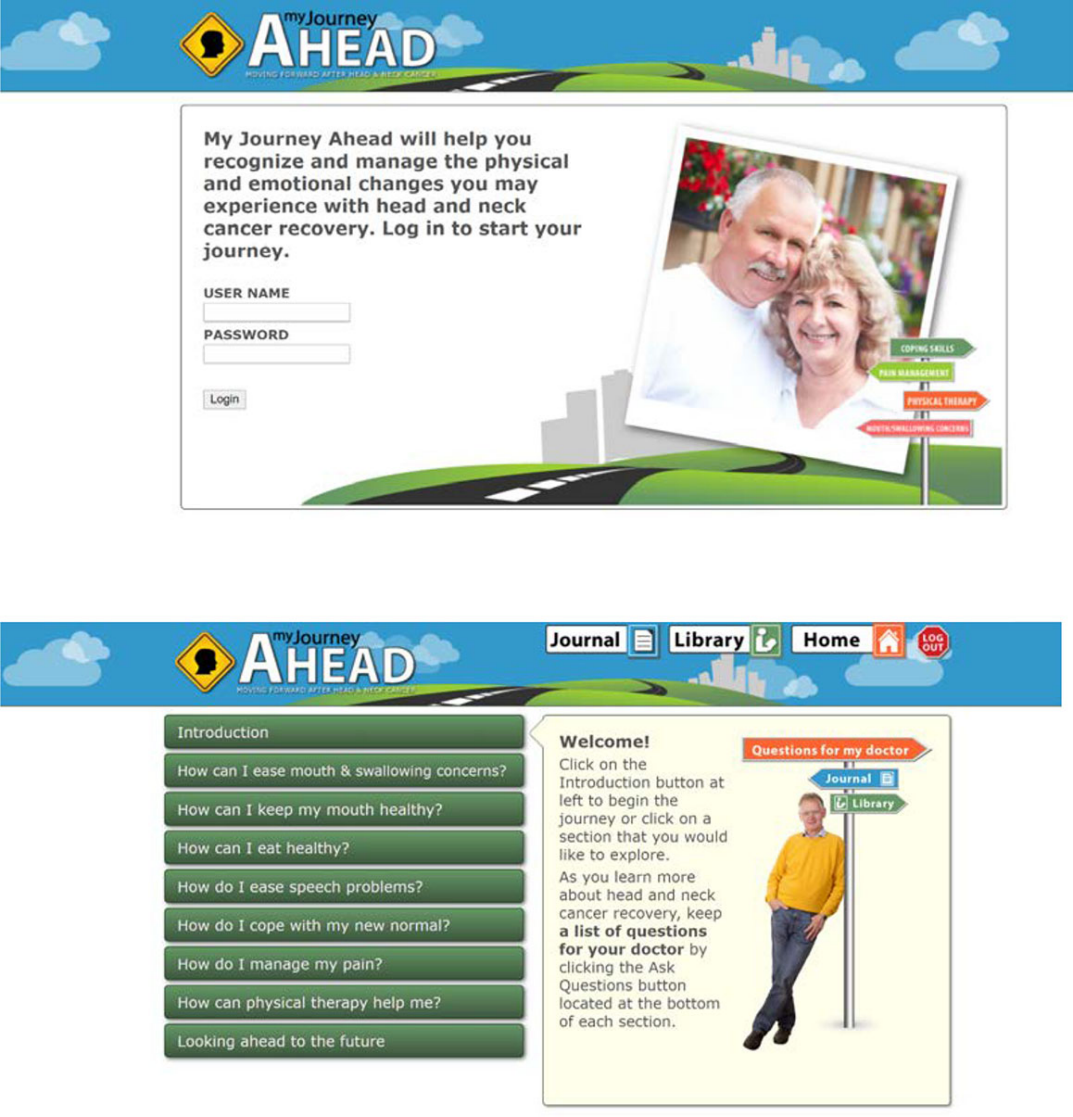
*My Journey Ahead* Login Screen and List of Units. Note: Images used in these screenshots were obtained from a public database.

Participants indicated that Unit 1 was not needed because all patients were already undergoing (or had completed) treatment. Therefore, participants believed that the information provided in this unit was not helpful or useful at this point in their cancer experience. Participants requested that we expand the content in Unit 2 pertaining to swallowing and oral care. As a result, the information in this unit was divided into separate units on mouth/swallowing concerns and oral health. With respect to the original Units 3 and 4, participants liked the content provided, but they requested additional examples and exercises. Finally, several participants noted that the program did not contain any information pertaining to physical therapy, nutrition or healthy eating, and pain management. Therefore, the revised program website contained an Introduction section, followed by seven units (instead of the initial four units) and a concluding section entitled “Looking Ahead to the Future” (see **Figure 1**). In addition, the program contained a journal feature (which is part of the unit on Coping with Cancer) and a library with various resources including program worksheets and videos, recipes and cookbook suggestions, and links to other websites (e.g., National Cancer Institute, American Cancer Society, Oral Cancer Foundation, etc.).

### Participants

We recruited a separate sample of patients to evaluate the revised website. Eligible participants included any patient with squamous cell carcinoma of the oral cavity, oropharynx, hypopharynx, or larynx who was currently undergoing or had completed either definitive or adjuvant radiation therapy within the past 12 months. Exclusion criteria included: 1) inability to read and/or communicate in English; 2) diagnosis of a head and neck cancer of non-squamous histology (e.g., adenoid cystic carcinoma, acininc cell carcinoma, adenocarcinoma, sarcoma); 3) blindness or severity of visual impairment that precluded one’s ability to view images/text; and 4) inability to provide informed consent.

### Procedures

Eligible patients were identified by collaborating clinicians and approached by a study coordinator who described the study procedures and obtained written informed consent. Following consent, study participants completed a baseline assessment and were then provided with a unique login ID and password for accessing the website. Participants could view the program at home, or in a private clinic space if they did not have Internet access or a web-enabled device. Participants who had not logged into the program after one week were contacted by the study coordinator who offered assistance and answered any questions. After 3 reminder calls, participants who had not logged in to the program were considered to have passively dropped out of the study. Participants who viewed the program were contacted approximately two weeks after their initial login to complete the follow-up assessment.

### Measures

Study assessments were collected at two time points: (1) at study entry and (2) two weeks after viewing the web-based program.

#### Demographic and Medical Variables

Participant characteristics including age, gender, race and ethnicity, education, annual household income, and marital status were assessed at baseline. Disease and treatment-related variables including: tumor site, disease stage, time since diagnosis, treatment(s) received, and time since treatment end were extracted from the electronic medical record by research staff.

#### Self-Efficacy

At both baseline and post-program assessment, self-efficacy for coping with cancer-related stressors was assessed using the brief version of the Cancer Behavior Inventory (CBI-B) (20, 21), a 12-item shortened version of the original CBI. The CBI-B is a validated measure of self-efficacy for coping with the major issues faced by people with cancer. For example, items measure an individual’s level of confidence in being able to maintain independence and a positive attitude; seek and understand medical information; cope with treatment-related side effects; and manage one’s affect. Each item is rated on a nine-point Likert-type scale, ranging from “1 = Not at all confident” to “9 = Totally confident”. Responses are summed to create a total score, which can range from 12 to 108, with higher scores reflecting greater self-efficacy in coping. In the study sample, Cronbach’s alpha for the CBI-B was 0.85.

#### Psychological Distress

Participant distress was measured at baseline and post-program using the Brief Symptom Inventory-18 [BSI-18 (22)], an 18-item instrument that has been used extensively in cancer patient populations (23, 24). The scale evaluates psychological symptoms during the past seven days. Participants are asked to rate the presence of each symptom on a scale from “0 = not at all” to “4 = extremely”. Responses are summed to yield a total global severity index (GSI), with higher scores representing greater levels of distress. As recommended by the scale developers, BSI scores were converted to standardized T-scores (22). In this sample, the Cronbach’s alpha for the overall global severity index was 0.89.

#### Program Evaluation

In the post-program assessment, participants were asked to evaluate their level of satisfaction with the program website and the information provided on a scale from 1 to 5, with higher ratings reflecting greater satisfaction. In addition, participants rated the usefulness of each unit on a scale ranging from “1 = Not useful” to “5 = Extremely useful”. Finally, participants provided overall ratings of the website (from “1 = Poor” to “5 = Excellent”) and how informative it was (“1 = Not informative” to “5 = Extremely informative”). An open-ended question was included at the end to allow participants to provide additional comments as desired.

### Analytic Strategy

The primary objective of this study was to characterize participant usage of and satisfaction with the web-based program. Descriptive statistics were used to characterize the participant sample, program usage, and levels of satisfaction with the program. Indicators of acceptability included: (1) A high degree of satisfaction with the information presented (mean ratings of 4 or greater on a 5-point rating scale); (2) high reported ease of use (mean ratings of 4 or greater on a 5-point scale); and (3) Overall rating (mean rating of 4 or greater on a 5-point scale).

To provide information relevant to the further development of the program, we also explored whether patient-level factors (gender, age, education level, time since diagnosis, treatment received) were associated with participant ratings or time spent exploring the website. Additionally, we explored potential changes in participant self-efficacy or distress after viewing the web-based program. Pearson’s correlations or simple linear regressions were used for hypothesis testing. The study was powered to have 80% power to detect correlations of 0.40 with 45 participants. This assumed a 5% Type I error rate (two-sided).

## Results

### Participant Characteristics

**Figure 2:**
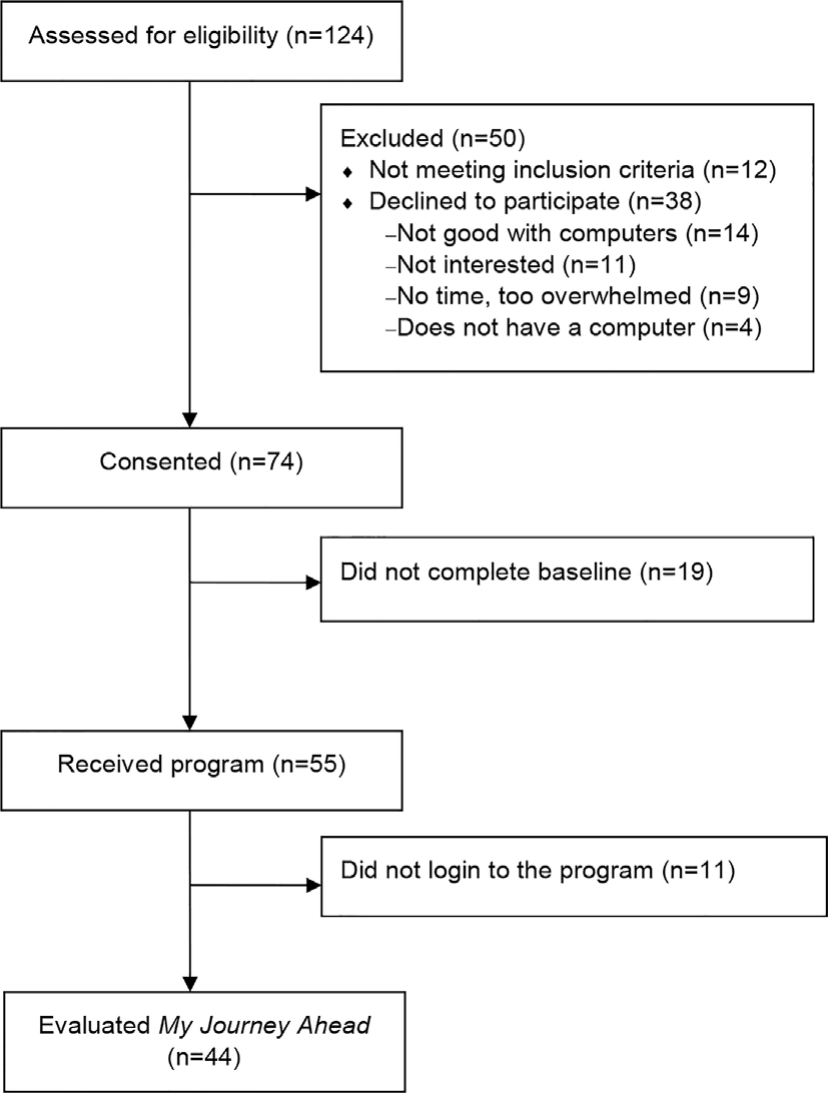
CONSORT Flow Diagram.

The CONSORT diagram is presented in **Figure 2**. Of the 124 patients assessed for eligibility, 12 did not meet inclusion criteria, 38 declined to participate, and 74 (66%) consented to enroll in the study. Of the 74 who consented to participate, 55 patients completed the baseline survey and were provided with a link to the web-based program and a participant-specific login number and password. Therefore, the present study sample includes 55 participants who had access to the program.

Participant characteristics are reported in **Table 1**. Participants were, on average, 61 years of age. The majority were male (75%) and married or living with a partner (82%). Over one-third of participants (36%) had completed a high school education or less, 53% had received some college education or a college degree, and 11% had obtained a post-graduate degree. Primary cancer site was predominantly oral cavity (46%) or oropharynx (35%) cancers. Most participants (93%) had access to a computer in their home.

**Table 1:** Participant Demographic and Clinical Characteristics (n = 55).

Variable	No. of participants (%)	Mean (SD)
Age (years)		61.1 (9.76)
Gender		
Male	41 (74.5%)	
Female	14 (24.5%)	
Marital status^a^		
Married/living as married	45 (81.8%)	
Single	3 (5.5%)	
Divorced/widowed	5 (9.1%)	
Education		
High school or less	20 (36.3%)	
Some college/college degree	29 (52.8%)	
Post-graduate degree	6 (10.9%)	
Annual household income^a^		
< $30,000	6 (10.9%)	
$30,000–$64,000	17 (30.9%)	
> $65,000	30 (54.5%)	
Access to computer at home	51 (92.7%)	
Primary cancer site		
Oral cavity	25 (45.5%)	
Oropharynx	19 (34.5%)	
Paranasal sinus	4 (7.3%)	
Larynx	3 (5.5%)	
Nasopharynx	1 (1.8%)	
Other	3 (5.5%)	
Disease stage		
1 or II	7 (12.7%)	
III or IV	48 (87.3%)	
Treatment(s) received		
Radiation only	6 (10.9%)	
Chemotherapy and radiation	17 (30.9%)	
Surgery and radiation	30 (54.5%)	
Surgery, chemotherapy, and radiation		
Time since diagnosis (months)	5.09 (2.23)	
Currently receiving radiation therapy	10 (18.2%)	
ime since treatment completed (weeks)^b^	8.23 (5.95)	

^a^The totals do not add up to 55 because 2 participants did not report marital status and annual household income. ^b^N = 45 patients who had completed treatment.

Greater than half of participants (55%) had undergone surgery and radiation therapy and 31% received chemoradiation treatment. Time since diagnosis was, on average, five months (SD = 2.23 months). The majority of participants (82%) had recently completed radiation prior to study enrollment [mean (M) = 1.9 months, standard deviation (SD) = 1.4 months], but 18% of the sample was still in treatment at the time of their participation. There were no differences in age, gender, marital status, education level, or income between participants who had completed treatment and those who were currently in treatment. In addition, no differences in self-efficacy in coping, global distress, program usage, or program evaluation ratings were observed between participants who had completed treatment and those still in treatment. Therefore, subsequent analyses are reported using the entire sample.

### Program Usage

Eleven of the 55 consented participants (20%) did not login to the website at all. Analyses indicated no differences in age, gender, marital status, education level, or income between those who visited the program website and those who did not (i.e. non-users). However, non-users reported higher baseline levels of cancer-specific distress (M = 36.7, SD = 7.8) compared with program users (M= 27.5, SD = 12.4), F(1,54)=5.51, *p* = 0.02. Non-users also reported slightly higher levels of global distress (M = 69.1, SD = 4.3) compared with program users (M = 66.5, SD = 4.0), F(1,54)=3.57, *p* = 0.06, and had slightly lower levels of self-efficacy in coping (M = 85.2, SD = 10.1) compared with program users (M = 93.0, SD = 13.8), F(1,54)=3.13, *p* = 0.08. Finally, non-users were more recently diagnosed (M = 3.8 months since diagnosis, SD = 1.8) compared with program users (M = 5.4 months, SD = 2.2), F(1,54)= 4.92, *p* = 0.03.

Among the 44 participants who visited the website, 13 logged in once and 15 logged in twice. The remaining 16 participants visited the website between 3 and 11 times. Among the 44 participants who visited the website, 27 (61%) viewed the Introduction and all seven units. Fifteen participants (34%) viewed between two and six units, and two participants viewed only the Introduction, but no additional content.

On average, participants spent a total of 68.5 min (SD = 55.3 min; range: 3–227 min) on the website, of which 62 min (SD = 56 min) were spent viewing the content contained in Units 1–7. The average length of time spent in each unit is presented in **Table 2**. Participants spent the longest time viewing the units on Coping (mean = 15.1 min, SD = 14.1 min), Nutrition (mean = 9 min, SD = 9.8 min) and Oral Health (mean = 7.61 min, SD = 6.43 min).

**Table 2:** Program Usage (n = 44).

Unit	# of Participants Visited	Average Minutes (SD)
Introduction	44	6.46 (4.71)
1-How can I ease mouth & swallowing concerns?	39	4.46 (3.53)
2-How can I keep my mouth healthy?	40	7.61 (6.43)
3-How can I eat healthy?	35	9.00 (9.78)
4-How do I ease speech problems?	33	5.14 (5.19)
5-How do I cope with my new normal?	35	15.09 (14.05)
6-How do I manage my pain?	32	7.25 (10.13)
7-How can physical therapy help me?	31	7.43 (15.81)
Looking Ahead to the Future	34	3.98 (3.90)
Total time		68.52 (55.30)

### Program Acceptability

Overall, participant feedback was positive (**Table 3**). On a scale of 1 to 5, where 5 represented the most positive rating, participants reported high levels of satisfaction with the information received (mean rating = 4.39), and agreed that they learned something new (mean rating = 4.06). Participants indicated that the information presented on the website was interesting (mean rating = 4.42) and of value (mean rating = 4.24). They also found the website easy to use (mean rating = 4.73) and the pictures and diagrams clear and understandable (mean rating = 4.58).

**Table 3:** Participant Evaluations.

Variable	Mean rating (SD)
**Ease of use and satisfaction^a^**	
Satisfied with the information received	4.39 (0.61)
Learned something new	4.06 (0.90)
Information was interesting	4.42 (0.66)
Website presented information that was valuable	4.24 (0.75)
Website was easy to understand	4.73 (0.45)
Pictures and diagrams were clear and understandable	4.58 (0.61)
**Unit-Specific Ratings^b^**	
1-How can I ease mouth & swallowing concerns?	4.06 (0.72)
2-How can I keep my mouth healthy?	4.29 (0.78)
3-How can I eat healthy?	4.10 (0.91)
4-How do I ease speech problems?	3.72 (1.02)
5-How do I cope with my new normal?	4.19 (1.06)
6-How do I manage my pain?	3.77 (0.97)
7-How can physical therapy help me?	3.97 (0.89)
**Overall rating**	
Overall, how informative was the website^c^	4.45 (0.75)
Overall rating of the website^d^	4.42 (0.61)

^a^On a scale from “1 = Strongly disagree” to “5 = Strongly agree.” ^b^1 = Not useful to 5 = Extremely useful”; ^c^1 = Not informative to 5 = Extremely informative; ^d^1 = Poor to 5 = Excellent.

With respect to usefulness, the program units that were most highly rated were Oral Health (mean rating = 4.29) and Coping with Cancer (mean rating = 4.19). The unit on Speech concerns received the lowest rating (mean rating = 3.72). When asked to rate the overall website on a scale of 1 = Poor to 5 = Excellent, participants’ mean rating was 4.42.

#### Patient Characteristics and Satisfaction

Satisfaction with the program was associated with certain patient characteristics. Older age was associated with higher levels of satisfaction (*r* = 0.48, *p* < 0.01). Higher baseline levels of self-efficacy in coping were associated with greater satisfaction (*r* = 0.36, *p* = 0.04) and higher overall ratings of the website program (*r* = 0.35, *p* < 0.05). In contrast, higher baseline levels of cancer-specific distress were associated with lower overall ratings (*r* = -0.35, *p* < 0.05). Higher baseline levels of global distress were also associated with lower program ratings (*r* = -0.59, *p* < 0.001) and with lower ratings of the information received from the website (*r* = -0.48, *p* < 0.01).

#### Program Usage and Participant Satisfaction

Participant satisfaction with the program was positively associated with program usage. Specifically, a greater number of program logins (*r* = 0.40, *p* = 0.02), more minutes spent logged in to the program website (*r* = 0.53, *p* < 0.01), and a greater number of units viewed (*r* = 0.46, *p* < 0.01) were each correlated with higher levels of satisfaction.

#### Change in Self-Efficacy and Distress

No significant changes were observed in self-efficacy in coping from baseline (M = 92.3; SD = 12.7) to post-program (M = 93.3; SD = 14.3). Similarly, global distress remained fairly stable from baseline (mean of standardized T scores = 67.0, SD = 4.13) to post-program (M = 66.2, SD = 4.8).

### Qualitative Feedback

Participants’ qualitative feedback in response to the open-ended item indicated that the information presented was reaffirming, and many patients noted that they especially valued the unit on coping:

“It helps to know that others have experienced the same difficulties and challenges, which is somewhat normalizing, and seeing examples of patients who have overcome many of these same challenges increases the likelihood of having a positive attitude when faced with rehabilitation and the hard work that comes with it.” - ID #1023

“It is nice to know how other people deal with stress and that it was normal.” - ID #1002

“It helped me to accept my limitations.” - ID # 1001

“[I] Do like the overall presentation and addressing an issue that does not get discussed.” – ID #1051

Participants had mixed responses to the unit on Speech Problems. While some participants felt that this unit was not relevant to them, others reported that it was helpful and provided unique perspectives.

“I liked the visual and audio example of a patient who obviously worked hard and found a way to communicate on his terms.” - ID #1009

“It gave insight [into] what other people deal with.” - ID #1055

“[It emphasized] to me the importance of patience in dealing with verbal deficiencies in social settings.” - ID #1058

Several participants mentioned viewing the program website with a family member or caregiver. The program was perceived to be helpful to others, not just patients.

“The information I already knew, [so] didn’t find it that useful. Personally [it] may help other family members to understand better.” - ID #1069

“We both feel that this site has a lot of potential to help others.” - ID #1060

Finally, one participant noted that he has not attended any support groups nor interacted with other patients, so the program was helpful for seeing how other patients have managed their cancer experience.

“I like knowing that you all care enough to have created the program at all. Not everyone has someone to be supportive.” - ID # 1009

Participant comments also provide guidance for how to improve program content and delivery in future versions. For example, a number of participants noted that this information would have been helpful to have earlier in the process, such as during treatment. Several noted that they would like more information on healthy eating, food choices, and swallowing problems. One commented that since individuals may have different needs, the ability to personalize the program would be useful. Finally, one participant would have liked to see slightly younger patients in some of the videos.

## Discussion

We developed a web-based program, *My Journey Ahead*, to provide information and strategies for managing concerns commonly experienced by HNSCC patients treated with radiation therapy. Participant ratings indicated that the program achieved its acceptability goals. Specifically, participants reported a high degree of satisfaction (i.e. mean ratings of 4 or higher on a 5-point scale) with the information presented, and they found the program to be interesting and of value. Most participants (70%) visited the program more than once, and 61% viewed all of the units. Participants spent the most time viewing the units on coping, nutrition, and pain. Overall feedback was positive, and participants found the web-based platform informative and easy to understand.

However, assessment of the program varied by patient characteristics. Specifically, older patients reported greater satisfaction with the program and were more likely to find the information presented to be of value. It is possible that younger patients are more facile with the internet, and thus, more able to find the information that they need (25, 26); whereas the current web-based program offered a valuable resource for older patients by providing reliable, evidence-based information in one easily accessible site.

Compared to the patients who used the program, those who did not log on (non-users) reported slightly higher levels of psychological distress and lower levels of self-efficacy in coping at study entry. Non-users were also more recently diagnosed, compared to program users. Interestingly, patients who engaged with the program indicated that having access to it earlier in their cancer experience would have been helpful. Patients may benefit from having access to this program when they are in the midst of undergoing challenging treatment regimens, but it is also a time when patients may feel least inclined or able to engage in such activities. A potential compromise may be to offer a specific unit each week, rather than providing all program content at once, which could feel intimidating and overwhelming to patients. Since each unit could viewed in a shorter time frame (e.g., approximately 10–15 min), this would help minimize patient fatigue and burden. Alternatively, patients may benefit from having access to the website earlier in their cancer treatment or before their radiation treatment begins.

Levels of distress and self-efficacy in coping did not change from pre to post-program. Because participants spent, on average, only one hour viewing the program, this was not likely to result in any significant impact on those endpoints. Most patients viewed the program in its entirety in either one or two sessions. It may be more optimal to design future iterations of the program so that the information and activities are distributed over time. For example, patients might be instructed to view one unit per week and provided with activities and exercises that they could practice or utilize each day during this period. Incorporating multiple opportunities for patients to apply the skills and tips suggested earlier in their treatment may result in greater benefit in self-efficacy over time.

Patients also provided helpful feedback regarding aspects of the program that could be enhanced. Some patients noted that they would like more information on eating, due to the considerable challenges they face with regard to eating and swallowing. One patient noted that since needs are individualized, any ability to personalize the program would be useful, and this may be especially true for younger patients. Similar to other programs developed previously, HNSCC patients are eager to have access to high-quality, evidence-based information to facilitate their recovery. For example, an educational telehealth intervention (utilizing a device attached to the user’s phone line) that was designed to promote symptom management was well-accepted and regularly utilized by HNSCC patients (27). Badr and colleagues developed a web-based self-management program for oral cancer survivors and their family caregivers to improve survivor self-management and QOL among both survivors and caregivers (28), which was well-received and demonstrated that survivors and caregivers are interested in using a web-based program. Thus, although a web-based program may not be as personalized as face-to-face support, technology-based interventions can ameliorate significant barriers to participation such as physical disability, geographic distance, or lack of providers/access.

We acknowledge several limitations of the present study. First, this was a single-arm study with a relatively modest sample size. Although there was no control group, the primary objective was to develop and evaluate the acceptability of a web-based program designed to provide information and strategies for managing symptom-focused concerns faced by HNSCC patients who were treated with radiation therapy. Patient responses indicated that the program was well-received and provided helpful information. We also gained useful insight regarding how to enhance program content and delivery. Second, due to the focus on program use and acceptability, other patient assessments were limited in order to reduce participant burden. As a result, only measures of psychological distress and self-efficacy in coping were collected at both study entry and follow-up. Subsequent larger studies should include additional patient-reported outcomes (e.g., symptom burden, quality of life), a longer follow-up time point, and measures of healthcare utilization. Finally, there may be key differences between those patients who viewed the program and those who did not. Our data suggest that patients who did not login to the website had higher levels of distress and lower levels of self-efficacy. Thus, identifying approaches for helping distressed patients obtain information and supportive services to address their needs represents an important element that should be incorporated in the next phase of this research. For example, patients who do not login to the program may benefit from referrals to other healthcare professionals, such as social workers or psychologists, who are trained to manage psychological distress and related issues. This approach is consistent with evidence-based stepped care programs, which have been demonstrated to be effective in reducing distress and improving quality of life among HNSCC patients (29).

In conclusion, our findings suggest that *My Journey Ahead* can serve as an informative resource for HNSCC patients who are undergoing or have recently completed radiation treatment. Further work should evaluate *My Journey Ahead* in a larger trial with a control group in order to explore potential effects of the program on patient self-efficacy in managing symptom-related concerns. Future studies should also incorporate appropriate strategies to address psychological distress in order to help patients thrive after treatment. Offering an easy-to-use web-based program, particularly for older patients who may have difficulty locating reliable evidence-based information on the internet, may increase information access and help address selected patient concerns.

## Data Availability Statement

The de-identified data reported in this article will be made available by the authors, without undue reservation.

## Ethics Statement

The studies involving human participants were reviewed and approved by the Fox Chase Cancer Center Institutional Review Board. The patients/participants provided their written informed consent to participate in this study.

## Author Contributions

CYF: conceptualization, methodology, formal analysis, investigation, resources, data curation, writing—original draft, writing—review and editing, supervision, project administration, and funding acquisition. TJG and BE: methodology, investigation, resources, and writing—review and editing. BLE: formal analysis, writing—original draft and writing—review and editing. JRB and JAR: validation and writing—review and editing. MC and JGB: resources and writing—review and editing. MLL: investigation, resources, and data curation. SLM: methodology and resources. CM: methodology, software, investigation, resources, data curation, visualization, project administration, and funding acquisition. All authors contributed to the article and approved the submitted version.

## Funding

This research was supported by the National Institutes of Health grants R41 CA144100 and P30 CA006927.

## Conflict of Interest

CM is employed by the company Triad Interactive, Inc.

The remaining authors declare that the research was conducted in the absence of any commercial or financial relationships that could be construed as a potential conflict of interest.
